# Acquired Vulvar Lymphangioma Circumscriptum

**DOI:** 10.1155/2013/967890

**Published:** 2013-12-16

**Authors:** Derya Uçmak, Sema Aytekin, Bilal Sula, Zeynep Meltem Akkurt, Gül Türkçü, Elif Ağaçayak

**Affiliations:** ^1^Department of Dermatology, Faculty of Medicine, Dicle University, 21280 Diyarbakir, Turkey; ^2^Department of Dermatology, Haydarpaşa Numune Training and Research Hospital, 34668 İstanbul, Turkey; ^3^Department of Pathology, Faculty of Medicine, Dicle University, 21280 Diyarbakir, Turkey; ^4^Department of Obstetrics and Gynecology, Faculty of Medicine, Dicle University, 21280 Diyarbakir, Turkey

## Abstract

Lymphangioma circumscriptum (LC) is a benign dilation of lymph channels localized to the skin and subcutaneous tissues. It is generally localized in mouth mucosa, tongue, proximal regions of arms and legs, groin, axilla, and trunk. Primary vulvar involvement is very rare. Vulvar involvement occurs in various clinical settings. Here, two uncommon cases with giant lymphangioma circumscriptum mimicking genital warts will be presented: a 55-year-old female patient with extensive lymphangiectasic lesions and genital wart-like papular lesions in the vulva secondary to diffuse scrofuloderma scars and a 60-year-old female patient with verruca-like lesions secondary to chronic inflammation.

## 1. Case 1

**Figure 1:**
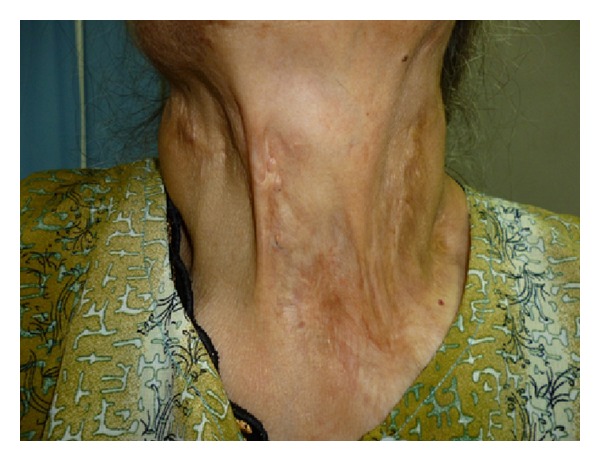
Scar tissue forming constrictions on the neck.

**Figure 2:**
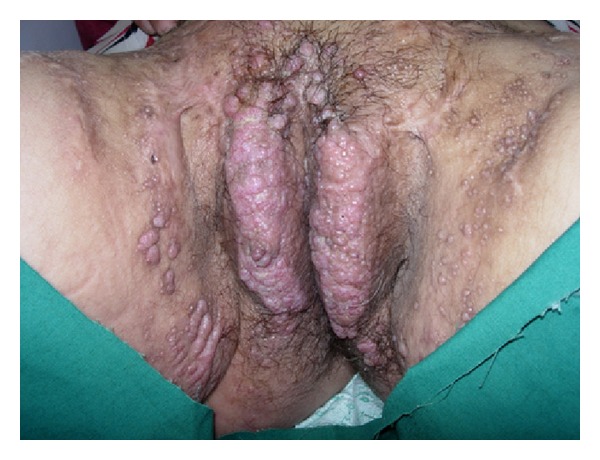
Widespread papules and vesicles on the pubis, labia majora, and inguinal region.

**Figure 3:**
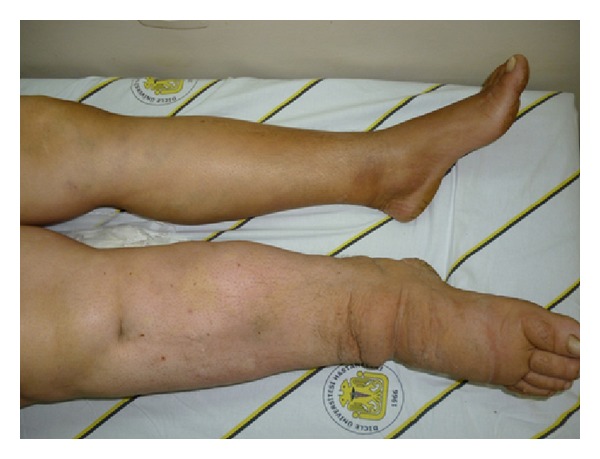
Nonpitting edema of the right leg and foot.

A 55-year-old female patient was admitted to our outpatient clinic for her complaints of oozy and itchy raised lesions. Dermatologic examination revealed multiple scars of scrofuloderma, typically in the form of a cord travelling over the neck, axilla, inguinal region, trunk, and the extremities (Figure 1). There was lymphedema of the vulva and labia majora associated with multiple bilateral pseudovesicular lesions which were a few mm in size. Some of the lesions were full of serous fluid and formed groups in the pubis, labia majora, vulva, and the inguinal region (Figure 2). There was obvious nonpitting edema in the right foot and leg (Figure 3).

History of the patient revealed exudative wounds which started in right side of the neck almost 35 years ago and spread over the axilla and to the bottom of the thorax and left scars as they healed. Similar lesions also occurred in the inguinal and genital regions almost 25 years ago. Doppler ultrasonography yielded normal results for this patient. All the other routine examinations including VDRL test produced normal or negative results. Histopathology of the lesions suggested lymphangioma. The patient whose complaints got very significant occasionally was recommended short term anti-inflammatory treatment for her exudative lesions in the vulvar region. In addition, the patient was referred to the plastic surgery outpatient clinic for a possible vulvectomy operation due to the severity of her symptoms. However, the patient rejected the operation and has been regularly followedup since then.

## 2. Case 2

**Figure 4:**
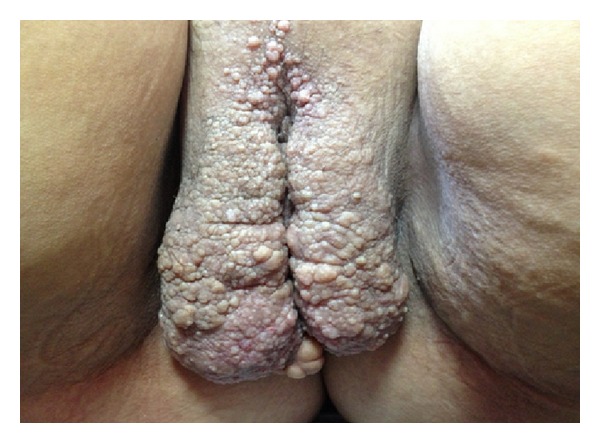
Large verrucous lesions covering the labia majora.

**Figure 5:**
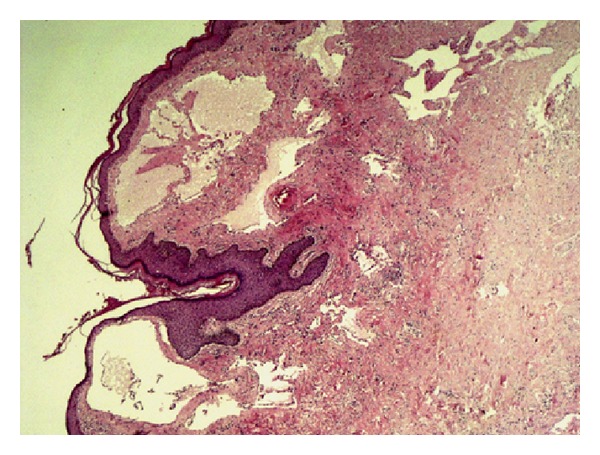
Dilated lymphatic channels containing eosinophilic proteinous material in the upper epidermis (HE, ×25).

A 60-year-old female patient presented to the gynecology and obstetrics outpatient clinic with complaints of abdominal distention. Ultrasonography suggested extensive ascites in the abdomen. Uterus and ovaries were not observed. The dermatology clinic was consulted about the verrucous lesions found in the genital examination of the patient (Figure 4), and skin biopsy was performed to confirm the prediagnosis of lymphangioma circumscriptum. Histopathology revealed large, irregular cystic dilatation of lymphatic channels consisting predominantly endothelial lining. These channels contain proteinaceous fluid and red cells in the upper dermis and lift up against the covering epidermis (Figure 5). For the purpose of exploring the etiology of the ascites, examinations were conducted on the patient whose history revealed that she underwent total abdominal hysterectomy and bilateral salphingo-oophorectomy 7 years ago. Peritoneal biopsy was taken from the patient to rule out malignancies. Peritoneal biopsy could not rule out tuberculosis, and the patient was considered to have a chronic inflammatory event. Ascites treatment was planned for the patient, and she was referred to the dermatologic surgery. This patient also rejected the surgery treatment. But, she has been visiting the clinic regularly for follow-up purposes since then.

## 3. Discussion

It is a frequently suggested hypothesis for the etiology of lymphangioma circumscriptum (LC) that superficial lymphatics of the skin cannot develop lymphatic connections in the deep layers. They are small clear herpetiform pseudovesicles [1]. Vesicles may be localized with clear borders or diffuse across a larger area or form groups. Primary lymphangioma stems from local malformations of lymphatics and manifests itself early in life. On the other hand, acquired lymphangioma develops secondary to chronic obstruction of lymphatics and can manifest itself at any age [2].

Primary vulvar involvement is quite rare and was found only in 73 cases until now in the literature. This appearance may mimic vulvar tumors [3]. Secondary lymphangioma of the vulva is a complication of pelvic lymphatic obstruction which occurs in the long term. Acquired lymphangioma occurs more commonly in the vulvar region compared to the other regions of the body, which can be frequently associated with surgery, radiation therapy, infection (erysipelas, tuberculosis, etc.), Crohn's disease, congenital dysplastic angiopathy, and congenital lymphedema [4]. A study with a large series of patients found a total of 12 cases with lymphangioma circumscriptum coming into existence after development of Crohn's disease and pelvic radiation exposure [5].

In the literature, there is a case with lymphangioma which followed Crohn's disease characterized by verrucous papules in the gluteal region [2]. Moreover, two other cases were initially diagnosed with genital warts when they developed LC in the vulva in 15 and 9 years, respectively, following hysterectomy and lymphatic node dissection which were conducted after radiotherapy of cervical cancer [6].

In addition, there is a pregnant case presented in the literature with lesions which clinically mimicked genital warts and actually stemmed from congenital deficiencies of the lymphatic system and occurred due to lymphatic circulation failure induced by pregnancy. At first glimpse, the patient was considered to have vulvar warts; however, detailed history taking and dermatologic and histopathologic examinations confirmed vulvar lymphangioma in the patient [7].

It is believed that scars of scrofuloderma do harm to the regional lymphatic system, paving the way for LC. The first case of ours was a patient with LC localized in the vulva, with scrofuloderma scars on the ground. As her lesions looked like verrucas, anogenital verruca was the initial consideration for this patient in the first examination. However, detailed history taking and examination processes revealed the presence of LC. The second case was a patient with lymphangioma lesions which developed secondary to a chronic inflammatory process in a similar mechanism to the previous one and were considered to be genital warts at first sight due to the hard papules.

The traditional treatment of LC—that is, surgical removal—is usually not successful due to rapid relapses and should be considered after treatment failures [8]. Vaporization with a CO_2_ laser is a recent recommendation for lymphangioma circumscriptum of the vulva and is suggested to yield acceptable cosmetic results [9]. Laser treatment and surgery were the options recommended to our patients, who rejected them both on the grounds that they had no cosmetic concerns.

Detailed dermatologic and histopathologic examinations should be performed on patients presenting with vulvar papular lesions for the possible coexistence of edema and pseudovesicles. Deep lymphatic ducts should be eradicated in order to avoid recurrences. Lymphangioma circumscriptum must certainly be considered in the differential diagnosis of verrucous lesions of the anogenital region.
